# Evaluation of anti-tetanus IgG antibody levels and influencing factors in patients undergoing hemodialysis

**DOI:** 10.3389/fimmu.2025.1678676

**Published:** 2025-12-03

**Authors:** Metin Özsoy, Hakkı Öztürk, Ayşegül Tuna, Artuner Varlıbaş, Salih Cesur, Altan Aksoy, Aydın Çifci, Mehmet Emin Demir

**Affiliations:** 1 Ankara Training and Research Hospital, Department of Infectious Diseases and Clinical Microbiology, Health Sciences University, Ankara, Türkiye; 2 Private Balgat Dialysis Center, Hemodialysis Physician, Specialist in Infectious Disease Epidemiology, Ankara, Türkiye; 3 Faculty of Medicine, Department of Infectious Diseases and Clinical Microbiology, Kırıkkale University, Kırıkkale, Türkiye; 4 Faculty of Medicine, Department of Internal Medicine, Kırıkkale University, Kırıkkale, Türkiye; 5 Department of Medical Microbiology, Ankara Bilkent City Hospital, Ankara, Türkiye; 6 School of Medicine, Medicana Ankara Hospital, Atılım University, Ankara, Türkiye

**Keywords:** hemodialysis, tetanus, vaccine immunity, anti-tetanus IgG, immunocompromised hosts

## Abstract

**Aim:**

This study aimed to assess anti-tetanus IgG antibody levels and identify determinants of inadequate tetanus immunity among maintenance hemodialysis (HD) patients.

**Methods:**

In this cross-sectional study, anti-tetanus IgG levels were measured by quantitative ELISA in 162 adult HD patients from two dialysis centers in Ankara, Turkey. Protective immunity was evaluated using both international (≥ 0.1 IU/mL) and robust (≥ 0.5 IU/mL) cut-offs. Demographic and clinical factors associated with immunity were analyzed by multivariate logistic regression.

**Results:**

Only 16.7% of HD patients achieved robust protection (≥ 0.5 IU/mL), whereas 49.8% had minimal protection (≥ 0.1 IU/mL). Protective immunity was independently associated with younger age (OR 1.07 per year; p = 0.004), shorter dialysis duration (OR 1.07; p = 0.030), male sex (female OR 2.92; p = 0.048), and recent booster vaccination within 10 years (OR 0.11; p < 0.001). Diabetes mellitus was not an independent factor.

**Conclusion:**

Most HD patients lacked durable tetanus immunity, particularly older females on long-term dialysis. The findings highlight the need for regular antibody monitoring, early revaccination, and structured booster programs to maintain adequate protection in this high-risk population.

## Introduction

Tetanus is a severe, toxin-mediated infection caused by *Clostridium tetani*, characterized by neuromuscular dysfunction and high mortality (1). Protection relies on adequate anti-tetanus IgG antibodies, achieved through vaccination (2, 3). Routine childhood immunization and 10-yearly boosters are recommended to sustain tetanus protection in the general population (2–4). However, immunity often declines with age, and adherence to booster schedules is suboptimal, leaving many adults susceptible (5, 6). Patients with end-stage renal disease (ESRD) on hemodialysis (HD) are particularly vulnerable due to uremia-related immune dysregulation (7), which impairs responses to infections and vaccinations (7, 8). ESRD patients typically have lower seroconversion rates and faster antibody decline after vaccines like hepatitis B and influenza, compared to healthy individuals (9, 10).

Despite tetanus prevention’s importance, studies in HD patients are limited. Available data indicate significantly reduced seroprotection rates among these patients. Sagheb et al. reported only 24% protection in Iranian HD patients versus 48% in healthy controls (11). Similarly, Krüger et al. observed initial protective immunity in just 44% of German dialysis patients, with only 38% responding to booster doses (12). Surveys show many adults do not receive recommended tetanus boosters, despite Advisory Committee on Immunization Practices (ACIP) guidelines (13, 14). Moreover, HD patients lose immunity rapidly; only ~32% maintain protective antibody levels five years post-vaccination (15). In international guidelines, the minimum protective antibody level is defined as ≥0.1 IU/mL (13, 16). However, several immunological and nephrology studies have indicated that higher titers (≥0.5 IU/mL) are associated with more durable and long-term protection in immunocompromised populations (12, 15, 17). Therefore, in this study, we evaluated both thresholds to better reflect the functional immunity status of HD patients.

In Turkey, data on tetanus immunity among HD patients is limited. While adult vaccination programs exist, their effectiveness in chronic disease patients is unclear. Thus, we assessed anti-tetanus IgG levels in a cohort of maintenance HD patients in Ankara, Turkey, identifying demographic and clinical factors linked to inadequate immunity. We compared our findings to published healthy control data, aiming to quantify the immunity gap and recommend strategies to improve tetanus protection in this high-risk group.

## Materials and methods

### Study design and population

This cross-sectional observational study assessed tetanus IgG immunity in 162 adult maintenance hemodialysis (HD) patients from two dialysis centers (Çankaya and Balgat, Ankara, Turkey). Patients aged ≥18 years and on HD ≥3 months were included; acute renal failure and peritoneal dialysis patients were excluded. Exclusion criteria included acute renal failure, peritoneal dialysis, history of organ transplantation, active malignancy, autoimmune disease, or use of long-term immunosuppressive therapy (≥3 months). None of the patients were on chronic corticosteroids or cytotoxic agents beyond routine dialysis-related medications. Baseline anti-tetanus antibody levels were measured without intervention. The study followed the Declaration of Helsinki guidelines, received local ethics committee approval.

### Data collection

Demographic and clinical data were obtained from medical records and patient interviews, including age, sex, dialysis vintage (duration), dialysis frequency (twice-weekly, thrice-weekly, or rarely four-times-weekly), body mass index (BMI, kg/m²), and comorbidities (diabetes mellitus, hypertension, cardiovascular disease, malignancy history). Immunosuppressive therapy use was noted, though none received long-term immunosuppressants beyond routine dialysis medication. Tetanus vaccination history within the last 10 years was recorded based on patient recall or medical records; uncertain cases were categorized as having no recent booster.

### Anti-tetanus IgG measurement

Pre-dialysis blood samples were collected for serum isolation. Anti-tetanus IgG concentrations were measured using quantitative ELISA standardized to WHO international units (IU/mL). Protective immunity was defined as IgG ≥0.50 IU/mL; titers <0.50 IU/mL indicated non-protective immunity, with levels <0.10 IU/mL representing negligible protection and ≥1.0 IU/mL strong protection. Assay detection limit was 5.0 IU/mL; one result exceeding this was recorded as 5.0 IU/mL. All samples were processed identically with appropriate quality controls.

### Healthy control data

Healthy control subjects were not enrolled due to logistical limitations. Comparative seroprevalence data were obtained from published studies (Sagheb et al., Sotoodeh-Jahromi et al.) evaluating similarly aged healthy adults, providing context for interpreting HD patients’ immunity levels (11, 16).

### Statistical analysis

Data were analyzed using SPSS v26.0 (IBM Corp., Armonk, NY). Normality was assessed via Shapiro-Wilk test; age and dialysis vintage were approximately normal (reported as mean ± SD), whereas anti-tetanus IgG levels, being right-skewed, were presented as median (IQR). Categorical variables were summarized as counts and percentages. Continuous variables (protected vs. non-protected groups) were compared using independent samples t-test or Mann–Whitney U test; categorical variables were analyzed with chi-square or Fisher’s exact tests. Statistical significance was set at p<0.05 (two-tailed). Multivariate logistic regression identified independent predictors of non-protective immunity (<0.50 IU/mL), including age, sex, dialysis vintage, diabetes, and recent vaccination. Dialysis frequency was initially included but later excluded due to non-significance. Adjusted odds ratios (OR) with 95% confidence intervals (CI) are presented, and model fit assessed by Hosmer-Lemeshow test. Given the exploratory nature, results with p<0.05 were interpreted cautiously without adjustments for multiple comparisons.

## Results

Patient characteristics: We analyzed 162 HD patients (mean age: 59.4 ± 10.5 years; median: 62, range: 25–75 years), with males comprising 62.3% (n=101). The majority were older adults; 75% were over 54 years, and 25% were over 67 years. Median dialysis vintage was 3.3 years (IQR: 1.6–7.7, range: 0.25–37 years). Most patients received thrice-weekly dialysis (85%), with 13% on twice-weekly and one patient on a four-times-weekly regimen. Mean BMI was 26 ± 6 kg/m² (median: 25, range: 18–52 kg/m²), indicating a wide spectrum from underweight to obese (**Table 1**).

**Table 1:** Baseline characteristics of the study population.

Characteristic	Value
Age, mean ± SD (years)	59.4 ± 10.5
Male sex, n (%)	101 (62.3%)
Dialysis duration, median (IQR), years	3.3 (1.6–7.7)
Dialysis frequency	
Thrice-weekly. Twice-weekly. Four-times-weekly.	137 (84.6%)
	21 (13%)
	1 (0.6%)
Body Mass Index, mean ± SD	26 ± 6 kg/m²
Diabetes mellitus, n (%)	62 (38.3%)
Hypertension, n (%)	111 (68.5%)
Malignancy history, n (%)	2 (1.2%)
Recent tetanus booster (<10 yrs)	17 (10.5%)

Comorbidities were common, including hypertension (68.5%) and predominantly type 2 diabetes mellitus (38.3%). Cardiovascular disease was not systematically documented but likely prevalent due to associated risk factors. Malignancy history was rare (1.2%), with no active chemotherapy or immunosuppressive therapy. ESRD causes varied (e.g., glomerulonephritis, diabetic nephropathy, hypertensive nephrosclerosis, polycystic kidney disease), but analysis focused broadly on comorbidities. Approximately 14 patients (8.6%) reported past traumatic injuries requiring medical attention (e.g., nail punctures, cuts, traffic accidents) from childhood through recent years (e.g., nail punctures around 1998, 2010; vehicle accidents in the 1970s–1980s; workplace injury in 2004). Most likely received tetanus boosters, though documentation was unclear.

Vaccination records were notably poor; only 17 patients (~10%) reported or documented tetanus immunization within the past 10 years, mainly due to injuries or pre-surgical protocols (2017–2020). Approximately 90% had either not received a booster for over a decade or could not recall adult vaccination. Older patients often reported their last vaccination during childhood or never receiving an adult booster; notably, three explicitly reported never having a tetanus shot as adults.

Anti-tetanus IgG levels were generally low (median: 0.10 IU/mL, IQR: 0.05–0.30 IU/mL) (**Table 2**). Half (50%) had negligible immunity (<0.10 IU/mL), and another 33.3% had non-protective levels (0.10–0.49 IU/mL) (**Table 2**). When using the ≥0.5 IU/mL threshold, only 16.7% (n=27) reached protective titers. However, when applying the internationally recognized ≥0.1 IU/mL threshold, 49.8% of patients were classified as having at least minimal protection. This dual-cutoff approach was adopted to distinguish between minimal and robust immunity, given the known accelerated antibody waning in HD patients. Only 10 patients (6.2%) exhibited strong immunity (≥1.0 IU/mL). The highest recorded titer (>5.0 IU/mL) was from a patient vaccinated two years prior.

**Table 2:** Distribution of anti-tetanus IgG titers and protection rates (n = 162).

IgG titer range (IU/mL)	Interpretation	n (%)
≥1.00	Strong protection	10 (6.2%)
≥0.50	Full protection (robust)	27 (16.7%)
≥0.10 to <0.50	Minimal protection	54 (33.3%)
<0.10	No protection (negligible)	81 (50.0%)
≥0.10 (combined)	Any level of protection (WHO)	81 (49.8%)
≥0.50 (combined)	Full protection (study threshold)	27 (16.7%)

An overwhelming majority of HD patients lacked adequate tetanus immunity. Even at the minimal protective threshold (≥0.10 IU/mL), usually seen in 70–85% of the general population, only 50% of HD patients reached protective titers, indicating substantially lower immunity levels.

**Figure 1:**
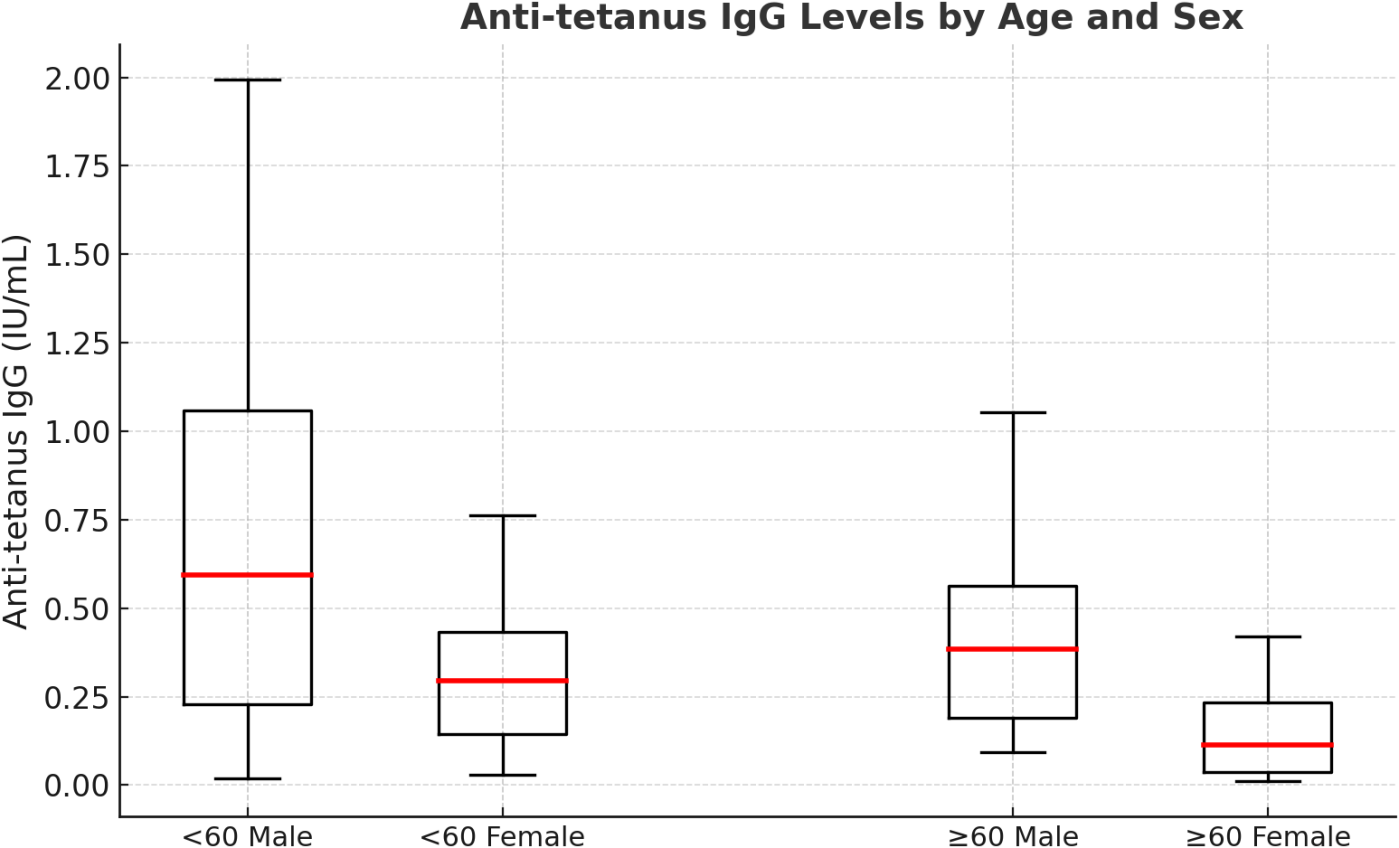
Boxplot of anti-tetanus IgG titer by age group (<60 vs ≥60 years) and sex. Figure shows that older patients tend to have lower median IgG levels than younger patients, and females tend to have lower levels than males.

Patients with protective antibody titers (≥0.50 IU/mL) were significantly younger (mean: 55.2 ± 9.4 years) than those without protection (mean: 60.3 ± 10.4 years, p=0.011), and predominantly male. Younger HD patients typically had higher IgG titers, while patients aged over 60 frequently showed very low antibody levels (**Figure 1**).

Sex differences were evident, with 18.8% (19/101) of males and 13.3% (8/60) of females showing protective antibody titers, a borderline statistical difference (p=0.055). Median IgG levels were slightly higher in males (0.11 IU/mL) than females (0.07 IU/mL), consistent with multivariate findings that females had lower immunity.

**Figure 2:**
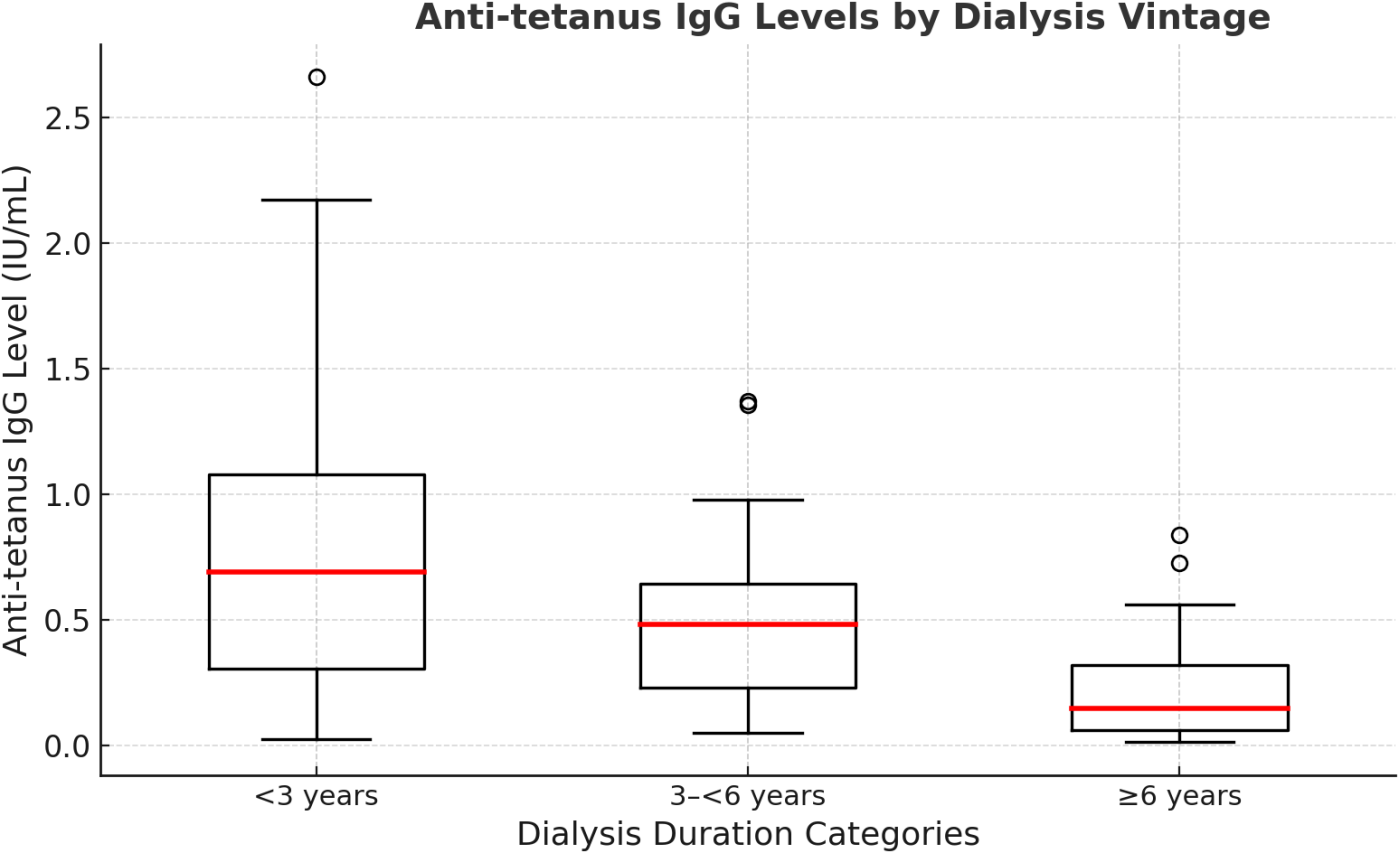
Anti-tetanus IgG levels according to dialysis vintage categories. Boxplots illustrating anti-tetanus IgG antibody titers among hemodialysis patients categorized by dialysis duration: <3 years, 3–<6 years, and ≥6 years. The figure demonstrates a progressive decline in median IgG levels with increased dialysis vintage, highlighting reduced tetanus immunity associated with prolonged dialysis duration.

Dialysis vintage inversely correlated with protective immunity. Patients with protective titers had shorter median dialysis durations (2.0 years) compared to non-protected patients (3.7 years, p=0.04). Only 8% (2/24) of patients dialyzed over 10 years had protective titers, compared to 27% (4/15) dialyzed under one year. **Figure 2** illustrates decreased IgG levels with increased dialysis vintage, suggesting prolonged dialysis and uremia reduce tetanus immunity.

Dialysis frequency (twice vs thrice weekly) was not significantly associated with immunity (14% protection in twice-weekly vs. 17% in thrice-weekly, p=0.75). The twice-weekly group was older with more diabetics, possibly confounding outcomes; thus, dialysis frequency alone had no significant effect.

**Figure 3:**
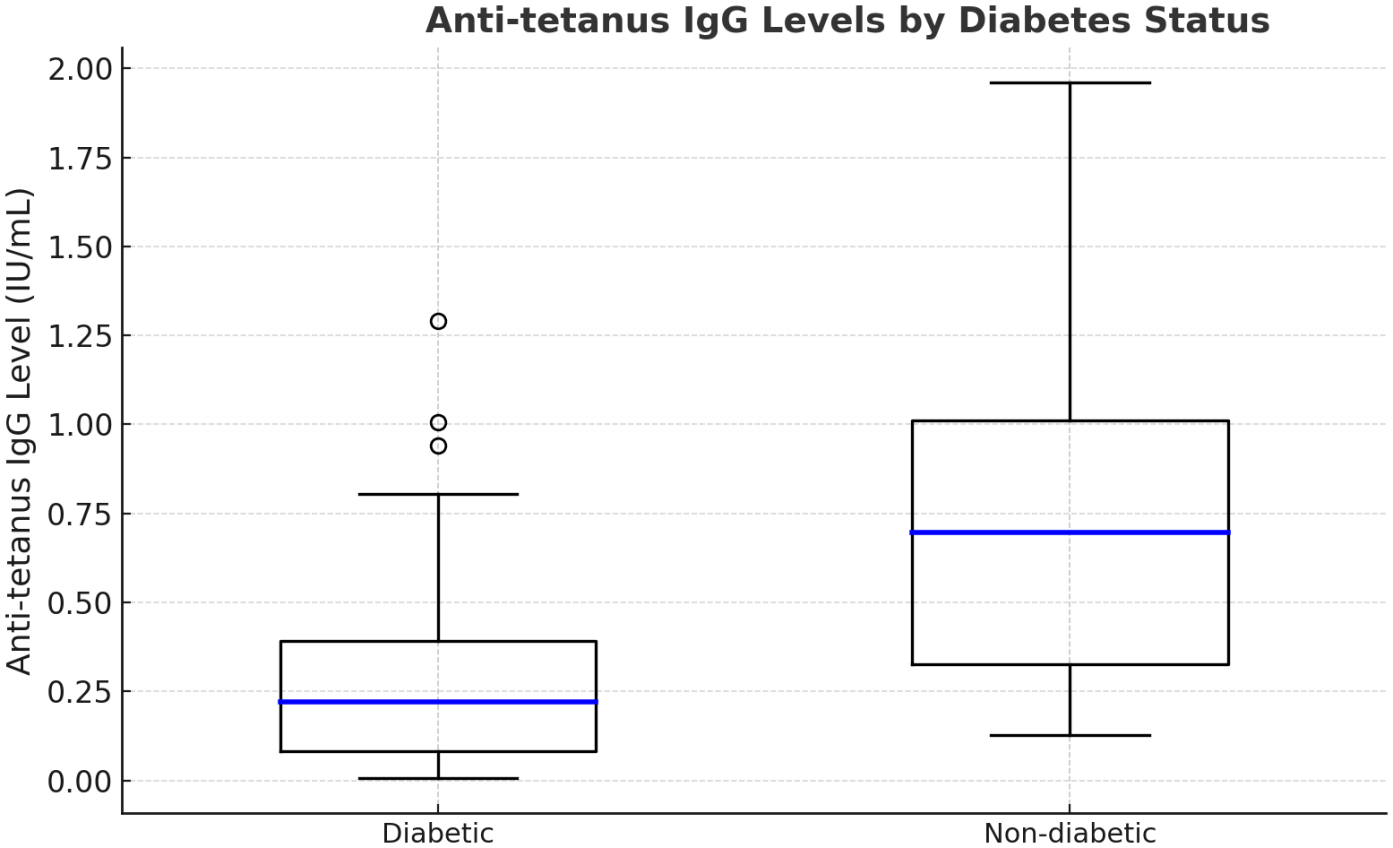
Boxplots illustrating anti-tetanus IgG antibody titers, comparing diabetic and non-diabetic patients undergoing hemodialysis. Diabetic patients tend to exhibit lower median IgG levels, suggesting that diabetes may contribute to reduced vaccine-induced immunity in this population.

Diabetic patients showed slightly lower protection rates than non-diabetics (12% vs. 19%, p=0.28; **Figure 3**), with mean IgG levels of 0.28 IU/mL (diabetic) vs. 0.35 IU/mL (non-diabetic). Hypertension, prevalent in about 67%, showed no effect on immunity. Diabetes was not independently significant after age adjustment.

BMI showed no linear relationship with tetanus titers; both low (<20) and high (>30) BMI groups had poor seroprotection, suggesting potential adverse effects from malnutrition or obesity. Most protected patients had mid-range BMI (20–30). However, BMI was excluded from regression analysis due to missing data and possible confounding factors like general health or albumin levels.

Recent tetanus vaccination strongly predicted immunity. Among patients with a booster in the past 10 years, 65% (11/17) had protective titers (≥0.50 IU/mL) compared to 11% (16/145) without recent vaccination (p<0.0001). Highest IgG titers occurred in recently vaccinated patients (e.g., 1.2, 2.3, >5 IU/mL from 2017–2018 boosters), whereas patients lacking boosters over 20 years had consistently low levels, highlighting the critical impact of recent vaccination.

Multivariate logistic regression (**Table 3**), adjusted for age, sex, dialysis vintage, diabetes, and recent vaccination, showed good model fit (Hosmer-Lemeshow p=0.47; c-statistic 0.78). Older age independently increased non-protective immunity odds by 7% per year (OR 1.07; 95% CI 1.02–1.12; p=0.004). Thus, every additional decade of age approximately doubled the odds of inadequate immunity, emphasizing age as a significant risk factor.

**Table 3:** Multivariate logistic regression predicting non-protective immunity (IgG < 0.50 IU/mL).

Variable	Odds ratio (OR)	95% CI	P-value
Age (per year increase)	1.07	1.02–1.12	0.004
Female sex	2.92	1.01–5.80	0.048
Dialysis duration (per year)	1.07	1.01–1.24	0.030
Diabetes mellitus	1.21	0.64–2.31	0.552
Recent booster vaccination (<10 yrs)	0.11	0.03–0.36	<0.001

Female sex independently predicted non-protective immunity, with females having nearly threefold higher odds compared to males (OR 2.92; 95% CI 1.01–5.8; p=0.048). Conversely, males had about 66% lower odds of non-protection (inverse OR 0.34), aligning with general population trends of lower female immunity due to fewer booster vaccination opportunities. Dialysis vintage independently increased odds of inadequate immunity by 7% per additional year (OR 1.07; 95% CI 1.01–1.24; p=0.030). Compared to 1 year of dialysis, odds of non-protective immunity increased approximately 1.14-fold at 3 years, 1.31-fold at 5 years, 1.60-fold at 8 years, 1.84-fold at 10 years, 2.58-fold at 15 years, and 3.62-fold at 20 years.

Diabetes mellitus did not significantly predict immunity (adjusted OR 1.21; 95% CI 0.64–2.31; p=0.552), likely due to balanced age distribution and similar representation (40%) among protected and unprotected groups. Recent tetanus vaccination within 10 years strongly predicted protection (OR 0.11; 95% CI 0.03–0.36; p<0.001), corresponding to a ninefold increase in odds of protective immunity, remaining significant after adjustment for other factors.

## Discussion

In this study of 162 hemodialysis patients, over 80% lacked protective tetanus immunity (anti-tetanus IgG <0.50 IU/mL), highlighting significant vulnerability in this population. Our finding of 16.7% seroprotection aligns closely with previous reports: Sagheb et al. observed 24% protection in Iranian dialysis patients (11), while Sotoodeh-Jahromi et al. found approximately 33% seroprotection (18). Differences may relate to our higher protection threshold (≥0.50 IU/mL) or variations in regional vaccination practices. Importantly, when applying the lower WHO-defined threshold of ≥0.10 IU/mL, nearly half of our cohort (49.8%) had detectable immunity, indicating that threshold selection significantly impacts reported protection rates.

Even after complete vaccination, dialysis patients exhibit suboptimal immune responses. One prospective study reported only 69% seroconversion following a full three-dose tetanus vaccine series in HD patients, versus nearly 100% in healthy controls (19). Such consistently low immunity rates indicate chronic kidney disease-related immunological impairment, paralleling poor ESRD patient responses to other vaccines (e.g., hepatitis B, influenza). Thus, earlier revaccination and routine antibody monitoring are recommended for HD patients. In previous comparative studies, Sagheb et al. reported tetanus seroprotection in 24% of Iranian hemodialysis patients compared with 48.2% among their healthy control group, whereas Sotoodeh-Jahromi et al. found protection rates of 34.6% in HD patients and 63.3% in matched controls. These findings highlight a consistent immunity gap between dialysis patients and healthy adults. However, such differences should be interpreted with caution, since vaccination schedules, booster adherence, and national immunization policies differ substantially across countries such as Iran, Turkey, and European nations (11, 18).

HD patients experience rapid antibody waning, making borderline or low titers insufficient for sustained protection. One European study found that while 96.5% of HD patients initially achieved protective titers post-vaccination, protection dropped sharply to 62% within six months (20). Krüger et al. similarly reported only 32% maintained protection five years post-vaccination, compared to the more prolonged immunity (≥10 years) typical in immunocompetent individuals (15). Although the World Health Organization defines ≥0.10 IU/mL as a minimal protective threshold, higher titers (e.g., ≥0.50 IU/mL) have been associated with longer-term protection and greater durability in high-risk groups, including HD patients (12, 17). Accordingly, we selected this more stringent threshold.

However, our data do not allow conclusions regarding vaccine responsiveness, as vaccination histories were mostly incomplete or absent, and when present, limited to a single booster. We therefore avoided inferring immunogenicity from antibody levels alone. Furthermore, while some statements suggest long-term waning of immunity, we acknowledge that longitudinal interpretation requires serial antibody measurements over time. Our findings represent a single cross-sectional snapshot, and future studies with follow-up titers are needed to confirm kinetics of antibody decay.

These observations are consistent with international findings, such as those by Girndt et al., who demonstrated reduced vaccine responsiveness among dialysis patients compared to healthy controls. In their study, 69% of HD patients developed protective titers after three vaccine doses, compared to 100% of healthy individuals, and HD responders had lower antibody levels (19). This impaired response likely explains our observed low seroprevalence long after initial immunization. Many patients initially received childhood vaccinations but failed to sustain adequate antibody levels due to ESRD-related immune impairment and lack of boosters. Long-term follow-up data further highlight this rapid antibody waning: only about one-third of vaccinated dialysis patients remain protected five years post-vaccination, suggesting the standard 10-year booster interval may be insufficient (15). More frequent boosters (e.g., every 5 years) and periodic antibody assessments might be necessary. Krüger et al. observed significantly higher 5-year mortality among dialysis patients who failed to respond to tetanus vaccination, potentially reflecting overall frailty and broader immune deficits. Although clinical outcomes weren’t tracked in our study, non-response to tetanus vaccination might indicate reduced general immune competence.

Multivariate analysis showed older age and prolonged dialysis vintage remained significant independent predictors of inadequate immunity after adjusting for vaccination status. The highest-risk profile identified was older female patients on long-term dialysis without recent vaccination, whereas younger males early in dialysis with recent boosters were most protected.

Age strongly influenced antibody levels; older HD patients exhibited significantly lower titers. This trend reflects both immunosenescence and historical gaps in booster uptake. Moreover, age-related antibody decline has also been documented in healthy adults, not only in HD patients (21, 22). Thus, lower protection in older adults may reflect both biological and policy-related contributors.

Sex differences in tetanus immunity likely reflect historical vaccination opportunities rather than biological factors. Adult men often receive boosters through military service or occupational health programs, while women typically only receive additional tetanus immunizations during pregnancy or proactively. In Turkey, for instance, young men receive a Td booster during compulsory military service, which most women miss. Our finding that female sex independently predicted non-protective antibody status aligns with Boey et al.’s Belgian study showing lower tetanus protection odds in women (23). Thus, targeted efforts to improve vaccination rates among female dialysis patients are warranted.

Dialysis vintage also independently correlated with reduced tetanus immunity. Extended duration of renal replacement therapy exposes patients to chronic uremic conditions, impairing immune function (e.g., T-cell dysfunction, chronic inflammation) (7, 24). Additionally, as the time since last vaccination increases, antibody waning becomes more pronounced. Despite frequent healthcare interactions, patients undergoing dialysis for many years often miss routine tetanus boosters unless prompted by specific injuries.

Previous studies similarly report declining tetanus antibody levels with longer dialysis duration (15, 25). Regular tetanus antibody monitoring and timely booster vaccinations, especially for long-term dialysis patients, could address this immunity gap.

In our analysis, diabetes mellitus was not independently predictive of tetanus immunity, despite general immune impairment associated with diabetes (26). Diabetic patients might have been younger on average, potentially masking diabetes effects. Nonetheless, maintaining up-to-date vaccination remains clinically crucial, given the increased infection risk in patients with CKD and diabetes.

BMI, used as a nutritional proxy due to inconsistent albumin data, did not show a clear linear relationship with antibody titers. Both low BMI (indicating malnutrition) and high BMI (potentially impairing immunity) could negatively impact immune function, suggesting a possible nonlinear relationship. Although our sample was insufficient to clarify this effect conclusively, addressing malnutrition and maintaining optimal nutrition likely benefits overall immune responsiveness in dialysis patients.

The most actionable finding of our study is the significant protective effect of recent tetanus booster vaccination. Patients vaccinated within the last 10 years exhibited high protection rates, whereas unvaccinated patients were mostly unprotected, strongly reinforcing current recommendations for decennial Td or Tdap boosters (27). This immunity gap likely occurs because adult tetanus immunization often gets overlooked in chronic illness management. Dialysis centers typically prioritize vaccinations directly relevant to ESRD (e.g., hepatitis B, influenza), leaving tetanus boosters dependent on primary care visits or emergency wound management. Many dialysis patients lack regular preventive healthcare, and some may mistakenly believe childhood vaccinations offer lifelong protection or remain unaware of booster requirements.

Notably, our findings demonstrate that patients with a recent booster dose had markedly higher antibody levels, supporting the continued immunogenicity of tetanus toxoid even in dialysis populations (27, 28). Although we did not assess vaccine responsiveness, our results support the benefit of recent booster vaccination. Given accelerated antibody decline in dialysis patients, experts suggest shorter intervals between boosters, potentially every 5 years, or periodic antibody titer monitoring to individualize booster timing (29). Additional booster doses for patients with suboptimal responses could be considered, akin to hepatitis B vaccination protocols in dialysis settings. Although routine adult tetanus titer monitoring is not currently standard, our findings support its consideration for high-risk groups, particularly long-term dialysis patients.

Targeted interventions should prioritize subgroups at highest risk. Elderly female dialysis patients and those on dialysis for over 5–10 years were least likely to have protective tetanus immunity, indicating they should be targeted for booster vaccinations and close follow-up. HD patients with uncertain or distant vaccination histories should also receive prompt boosters. Every healthcare encounter, such as wound care or hospital admission, presents an opportunity to update tetanus immunizations proactively, rather than relying solely on reactive administration after high-risk injuries. Regular vaccination would reduce vulnerability significantly.

## Conclusion

This study revealed that a substantial proportion of hemodialysis patients lack protective tetanus immunity, despite the preventable nature of the disease. Using the WHO-defined threshold of ≥0.1 IU/mL, 49.8% of patients had minimal protection, whereas only 16.7% achieved robust protection at ≥0.5 IU/mL. This suggests that many HD patients are inadequately protected, particularly against long-term or high-risk tetanus exposure. Factors significantly associated with inadequate immunity included advanced age, female sex, and prolonged dialysis duration, all common among dialysis patients. Booster administration within the past 10 years emerged as the single strongest modifiable predictor of protection. However, most patients had not received timely boosters, underscoring the need for systematic preventive strategies. This avoidable vulnerability persists despite regular healthcare encounters during dialysis treatments. As tetanus is entirely vaccine-preventable, dialysis providers should proactively ensure timely booster vaccinations. Routine immunization reviews or periodic antibody titer screenings, coupled with more frequent boosters as necessary, could significantly enhance protection in this high-risk group. Ultimately, bridging the immunity gap through structured vaccination programs may offer a simple yet highly effective strategy to protect dialysis patients from a completely preventable infection.

Our study has several limitations. First, we lacked a concurrent healthy control group, relying instead on external published data. These comparative data were drawn from cohorts in Iran and Germany, where vaccination policies and booster uptake may differ from those in Turkey. This geographical and policy-related variability should be considered when interpreting seroprotection differences. Second, vaccination histories were based on patient recall, which can be imprecise; however, antibody measurement provided an objective immunity marker. Third, our protective immunity threshold (≥0.5 IU/mL) might be debated, as some guidelines accept ≥0.1 IU/mL as protective. To address this, we included both thresholds in our analysis. While ≥0.1 IU/mL represents minimal protection per WHO guidance, ≥0.5 IU/mL reflects more robust and durable immunity based on prior studies in HD patients. Fourth, we only assessed tetanus-specific antibodies without evaluating diphtheria or other vaccine components; future studies should measure both tetanus and diphtheria immunity for a comprehensive assessment. Lastly, our findings from two urban centers in Turkey might not fully generalize elsewhere. Nevertheless, consistency with international reports (e.g., from Iran and Germany) suggests widespread applicability. Moreover, differing national vaccine schedules, laboratory assays, and timing of last vaccination could partly explain these discrepancies The inherent immune suppression from chronic dialysis likely places HD patients globally at risk of tetanus immunity loss unless actively revaccinated.

## Data Availability

The raw data supporting the conclusions of this article will be made available by the authors, without undue reservation.
